# Optimizing miRNA-module diagnostic biomarkers of gastric carcinoma via integrated network analysis

**DOI:** 10.1371/journal.pone.0198445

**Published:** 2018-06-07

**Authors:** Fengbin Zhang, Wenjuan Xu, Jun Liu, Xiaoyan Liu, Bingjie Huo, Bing Li, Zhong Wang

**Affiliations:** 1 The Fourth Hospital of Hebei Medical University, Shijiazhuang, China; 2 School of Life Sciences, Beijing University of Chinese Medicine, Beijing, China; 3 Institute of Basic Research in Clinical Medicine, China Academy of Chinese Medical Sciences, Beijing, China; 4 TCM Hospital of Hebei Province, Shijiazhuang, China; 5 Institute of Information on Traditional Chinese Medicine, China Academy of Chinese Medical Sciences, Beijing, China; 6 Shanxi Buchang Pharmaceutical Co.,Ltd., Xi’an,China; University of Massachusetts Medical School, UNITED STATES

## Abstract

Several microRNAs (miRNAs) have been suggested as novel biomarkers for diagnosing gastric cancer (GC) at an early stage, but the single-marker strategy may ignore the co-regulatory relationships and lead to low diagnostic specificity. Thus, multi-target modular diagnostic biomarkers are urgently needed. In this study, a Z_summary_ and NetSVM-based method was used to identify GC-related hub miRNAs and activated modules from clinical miRNA co-expression networks. The NetSVM-based sub-network consisting of the top 20 hub miRNAs reached a high sensitivity and specificity of 0.94 and 0.82. The Z_summary_ algorithm identified an activated module (miR-486, miR-451, miR-185, and miR-600) which might serve as diagnostic biomarker of GC. Three members of this module were previously suggested as biomarkers of GC and its 24 target genes were significantly enriched in pathways directly related to cancer. The weighted diagnostic ROC AUC of this module was 0.838, and an optimized module unit (miR-451 and miR-185) obtained a higher value of 0.904, both of which were higher than that of individual miRNAs. These hub miRNAs and module have the potential to become robust biomarkers for early diagnosis of GC with further validations. Moreover, such modular analysis may offer valuable insights into multi-target approaches to cancer diagnosis and treatment.

## Introduction

Gastric cancer (GC) is one of the most common malignancies and the second cause of cancer mortality worldwide, posing a major risk to public health [1–2]. Due to the difficulties in early diagnosis, the overall five-year survival rate after surgery is only 20%-30% in advanced-stage GC patients [3]. Although chemotherapy may improve the survival rate of GC patients after surgery, the efficacy of chemotherapy is limited by metastasis and drug resistance [4–6]. Several novel chemotherapeutic and molecular targeted agents, including irinotecan (CPT-11), taxanes, oxaliplatin, trastuzumab, sunitinib, and bevacizuma, have been used to improve the outcomes of GC patients, but the prognosis of advanced or recurrent GC still remains unsatisfactory [7]. Therefore, it is crucial to identify novel biomarkers for diagnosing GC at an early stage.

As with many other complex diseases, the occurrence of GC also owes to the disturbance of multiple genes at the global molecular network level [8–9]. The current single-target diagnostic or therapeutic strategies may ignore the interactions between several molecular targets and lead to a low efficacy. Therefore, multi-target modular network research may contribute significantly to more rational and effective diagnostic and therapeutic strategies [10]. A lot of network-based studies have attempted to find module biomarkers or targets of GC at gene expression, mRNA transcription or microRNA (miRNA) levels [11–12], but how to quantificationally identify GC-related activated modules as early diagnostic biomarkers still remains challenging.

MiRNAs are small non-coding RNAs of 18–24 nucleotides that regulate gene expression by mediating mRNA degradation or repression of mRNA translation [13]. It has been shown that miRNAs can serve as biomarkers, drug targets, or tumor suppressors by regulating the expression of cancer-related genes [14]. Many miRNAs have been found to be related with GC [15–16], and miRNAs may represent the bridge between Hp-related gastritis and GC development [17]. Although GC-related miRNA networks have been analyzed [18], there is still a lack of diagnostic miRNA module biomarkers for GC.

In this study, based on miRNA co-expression networks, Z_summary_ and Support Vector Machine (SVM) algorithms were used to systematically identify the hub miRNAs and activated modules that could potentially be used as GC biomarkers.

## Materials and methods

### Normal and GC miRNA expression datasets

The miRNA expression data of GC were obtained from Gene Expression Omnibus database (GEO) with accession number of GSE7390, which were originated from a study by Kim et al [19]. The custom-designed Agilent microarray contained 1,667 unique miRNA sequences across all species and each probe had 4 replicates. In this study, human-related unique miRNAs were selected. The miRNA expression data of 90 GC patients were used as the reference group (GC group, **S1 Table**), another replicated datasets of the same samples were used as the test group (Rep group, **S2 Table**), and the miRNA expression data from 34 healthy volunteers were considered as the normal control group (Norm group, **S3 Table**).

### Detection of miRNA co-expression modules

The construction of miRNA co-expression networks was implemented in the WGCNA R package [20]. A matrix of pairwise correlations was constructed between all pairs of miRNA probes by using appropriate soft-thresholding. And then, topological overlap measure (TOM) and Dynamic Hybrid Tree Cut algorithm [21] were used to detect the miRNA co-expression modules, and each module was assigned a color. Appropriate soft-thresholds were selected for each dataset when the network met the best scale-free topology criterion, and the minimum module size was set at 3.

### Module-based comparison among different groups

In order to compare the miRNA co-expression patterns at network module level, we used the module-based consensus ratio (MCR) to illustrate the module similarities across different groups [22]. The MCR was defined as the ratio of significantly overlapped module pairs to all the module pairs between two groups. The significantly overlapped module pairs were determined based on a Fisher's exact test (p < 0.05). The MCR is defined by Eq 1, where NM represents the number of modules, *a* and *b* represent two different groups, and NM_overlap_ is the number of module pairs with a p < 0.05.

$${\mathrm{MCR}}_{a,b}=\frac{{\mathrm{NM}}_{\mathrm{overlap}}}{{\mathrm{NM}}_{a}\times {\mathrm{NM}}_{b}}\times 100\%$$ (1)

### Identification of miRNA module biomarkers

We adopted a Z_summary_ statistic to identify the preserved and activated modules related to GC. The Z_summary_ value is composed of 4 statistics related to density and 3 statistics related to connectivity, which can quantitatively assess whether the co-expression patterns of a specific module in the reference group is preserved or disrupted in the test group. The equation of Z_summary_ is listed below Eq 2, where a Z_summary_ ≥ 2 indicates preservation or reproducibility, while a negative Z_summary_ value indicates disruption or variation [23]. Compared with the Norm group, modules with a negative Z_summary_ value in the GC group would be considered activated modules, which might potentially be used as the miRNA module biomarkers of GC.

$$Z_{\mathrm{summary}}=\frac{{\mathrm{median}(Z}_{\mathrm{meanCor}}{,}_{\mathrm{ZmeanAdj}}{,Z}_{\mathrm{propvarExp}\;l}{,Z}_{\mathrm{meamKME}})+{\mathrm{median}(Z}_{\mathrm{cor}.\mathrm{KIM}}{,Z}_{\mathrm{cor}.\mathrm{KME}}{,Z}_{\mathrm{cor}.\mathrm{cor}})}{2}$$ (2)

### SVM-based identification of core miRNAs and sub-network

In order to identify the core miRNAs and sub-network as potential GC biomarkers, the NETwork constrained Support Vector Machines (NetSVM) Cytoscape App was used in our study [24]. As an extension of the conventional SVM, NetSVM exploits the decision hyperplane to predict the classification of genes, which can be used to identify biologically meaningful network biomarkers from network and gene expression data. Along with the core nodes and sub-network, NetSVM also reports the sensitivity, specificity, ROC curve and AUC values for the classification. In our study, the GC miRNA expression data and network data were entered into the NetSVM Cytoscape App, and the top miRNAs and sub-network with higher weights were identified.

### Evaluation of the diagnostic performance of module biomarkers

Based on the expression level of the miRNAs in the GC and Norm groups, the area under the ROC curve (AUC) was used to evaluate the diagnostic performance of the miRNA module biomarkers. All analyses were performed using SPSS 19.0 software. For the modular biomarkers, the original single miRNAs’ expression value were combined, and the mean value was used for the ROC curve analysis. The NetSVM-based weights were taken into account when calculating the mean expression value of miRNA modules. The weighted value was used as the summary score for the diagnostic tests. Optimal cut-off value was set as the threshold with the highest Youden's index (Sensitivity + Specificity -1).

### Prediction of the target genes of miRNA modules

After the identification of GC activated miRNA modules, we predicted their target genes based on three independent databases, i.e. Targetscan Human 7.1 (http://www.targetscan.org/), miRDB (http://www.mirdb.org/miRDB/), and miRTarBase (http://mirtarbase.mbc.nctu.edu.tw/). The GC activated miRNA modules were loaded into the three databases and the background species was selected as “human”. Genes that were predicted by all of the three databases were considered as target genes for further analysis.

### Functional annotation of miRNA modules

To characterize the functions of activated miRNA modules and further investigate the mechanisms underlying the development of GC, we performed GO and KEGG pathway enrichment analysis by using the Database for Annotation, Visualization and Integrated Discovery (DAVID) [25]. For each module, an over-representation of a functionally relevant annotation was defined by a modified Fisher’s exact p-value with an adjustment for multiple tests using the Benjamini method. GO terms and pathways with a p < 0.05 were considered significant.

## Results

### GC miRNA co-expression network and modules

**Fig 1:**
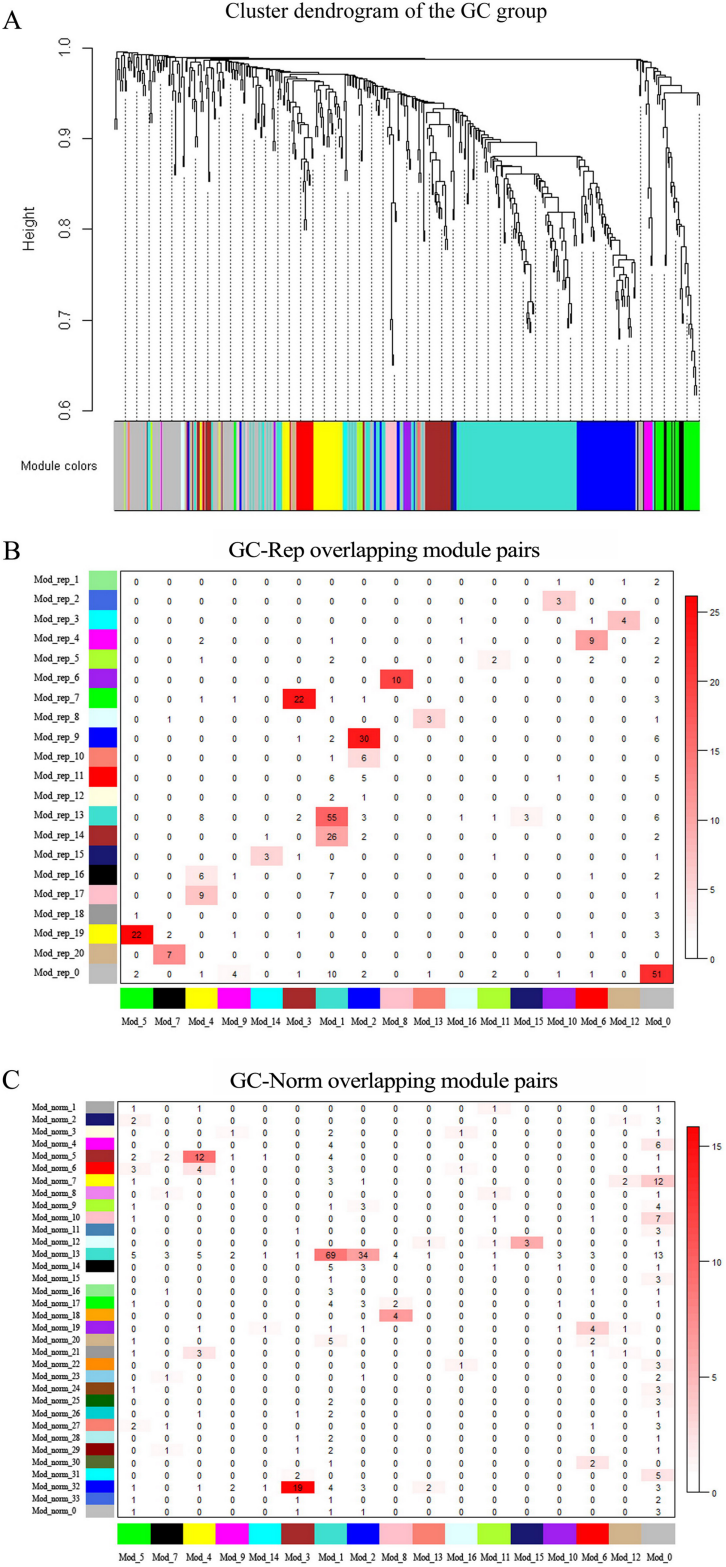
Hierarchical cluster modules of GC group and their similarities with those of Rep and Norm groups. **A**. The hierarchical cluster tree (dendrogram) of GC group; each major tree branch represents a module, and each module is labeled with a color below the dendrogram. **B**. Similarity of modules between GC and Rep groups; each row of the table corresponds to GC modules (labeled by color and labels), and each column corresponds to Rep modules. Numbers in the table indicate gene counts in the intersection of the corresponding modules of GC and Rep groups. Coloring of the table encodes -log (p), with p being the Fisher's exact test p-value for the overlap of the two modules. The darker the red color is, the more significant the correlation is. **C**. Similarity of modules between GC and Norm groups; the table legend is the same as (**B**).

As described in the Methods section, the WGCNA analysis was performed to construct the GC miRNA co-expression network and detect modules. Based on the scale-free topology model computation, the soft threshold power was set at β, of which was appropriate for the network construction (**S1 Fig**). The hierarchical clustering procedure found 16 miRNA co-expression modules, and each module corresponded to a branch of the resulting clustering tree and was labeled a unique color (**Fig 1A**). The network hotmap of all miRNAs is provided in **S2 Fig**. The detailed information about the miRNA module membership labeled by colors and numbers can be found in **S1 Table**. The average size (the number of miRNAs) of GC modules was 20, ranging from 3 to 120.

### Module similarities

To investigate the miRNA co-expression patterns of GC, Rep and Norm groups at network module level, we compared their module similarities based on MCR. There were 20 and 33 modules in the Rep and Norm groups, respectively. Compared with the Rep and Norm groups, the consistencies in miRNA composition and module pairs with a certain number of overlapping miRNAs in the GC group are presented in **Fig 1B and 1C**. It showed that the number of GC-Rep overlapping module pairs was much larger than that of GC-Norm overlapping module pairs. Almost all the miRNA modules in the GC group overlapped with those in the Rep group, particularly Mod_8 completely overlapped with Mod_rep_6, reflecting the reproducibility of modules. The MCRs for pairwise comparisons were 5.0% (GC vs. Rep) and 2.8% (GC vs. Norm), respectively, indicating significant variation of the miRNA co-expression pattern in GC patients.

### Z_summary_-based module preservation

**Fig 2:**
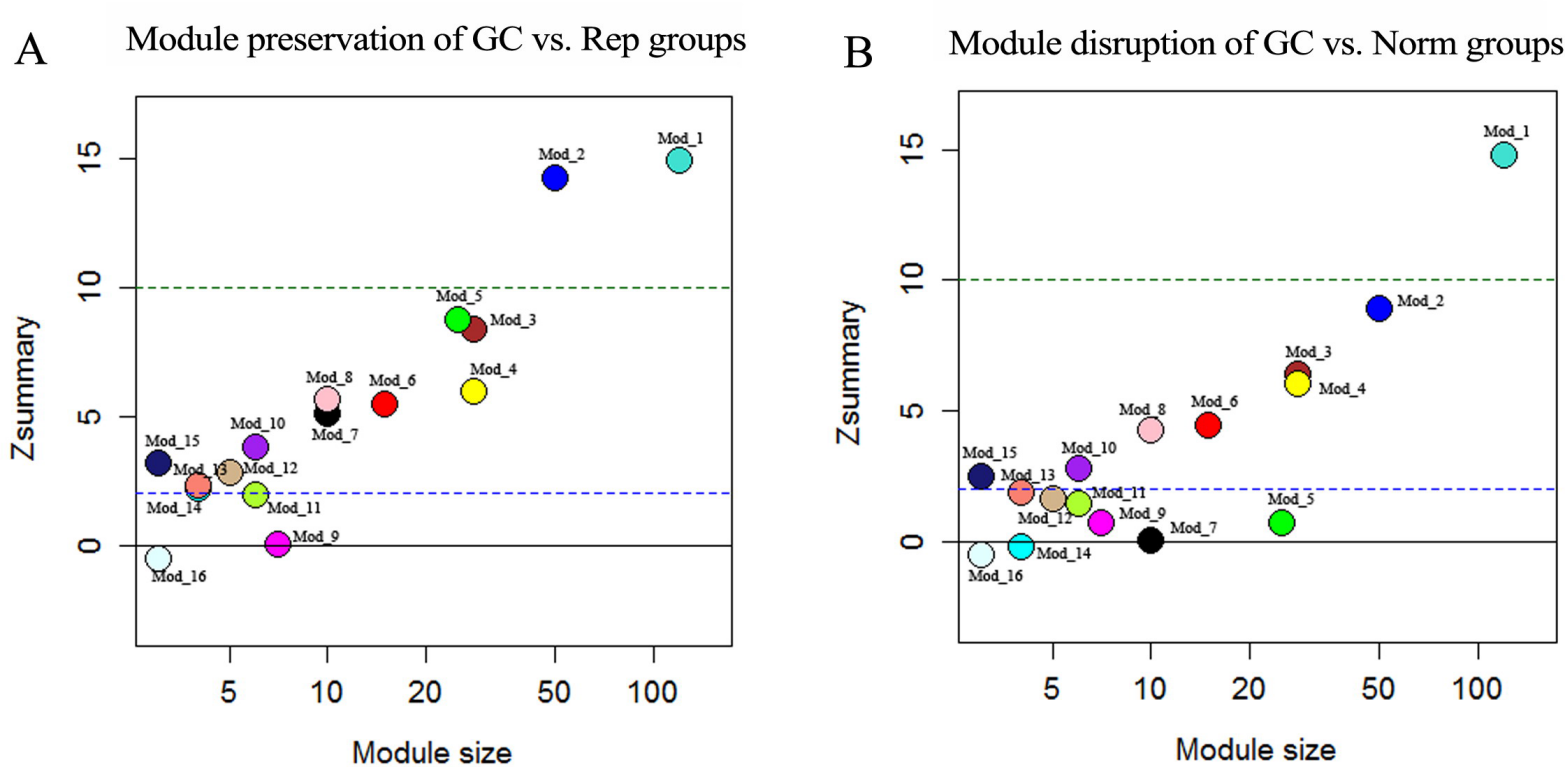
Z_summary_-based preservation and disruption of GC modules. **A**. The Z_summary_ preservation statistics (y-axis) of the GC modules compared with Rep group. The dashed blue and green lines (Z_summary_ = 2 and 10) indicate moderate and strong preservation thresholds, respectively. **B**. The Z_summary_ disruption statistics (y-axis) of the GC modules compared with Norm group. Modules with a negative Z_summary_ value are considered as activated modules.

In order to assess whether the miRNA modules in the GC group were preserved, the Z_summary_ values of each module were calculated with Rep as the test group. As mentioned above, a Z_summary_ ≥ 2 indicates module preservation, and a Z_summary_ ≥ 10 represents strong preservation [23] or module reproducibility. **Fig 2A** lists the Z_summary_ values of all the miRNA modules in the GC group compared with the Rep group. Except Mod_9, Mod_11, Mod_16, all the other modules were preserved, and two modules (Mod_1 and Mod_2) were strongly preserved in the GC group.

### Z_summary_-based module biomarker

**Fig 3:**
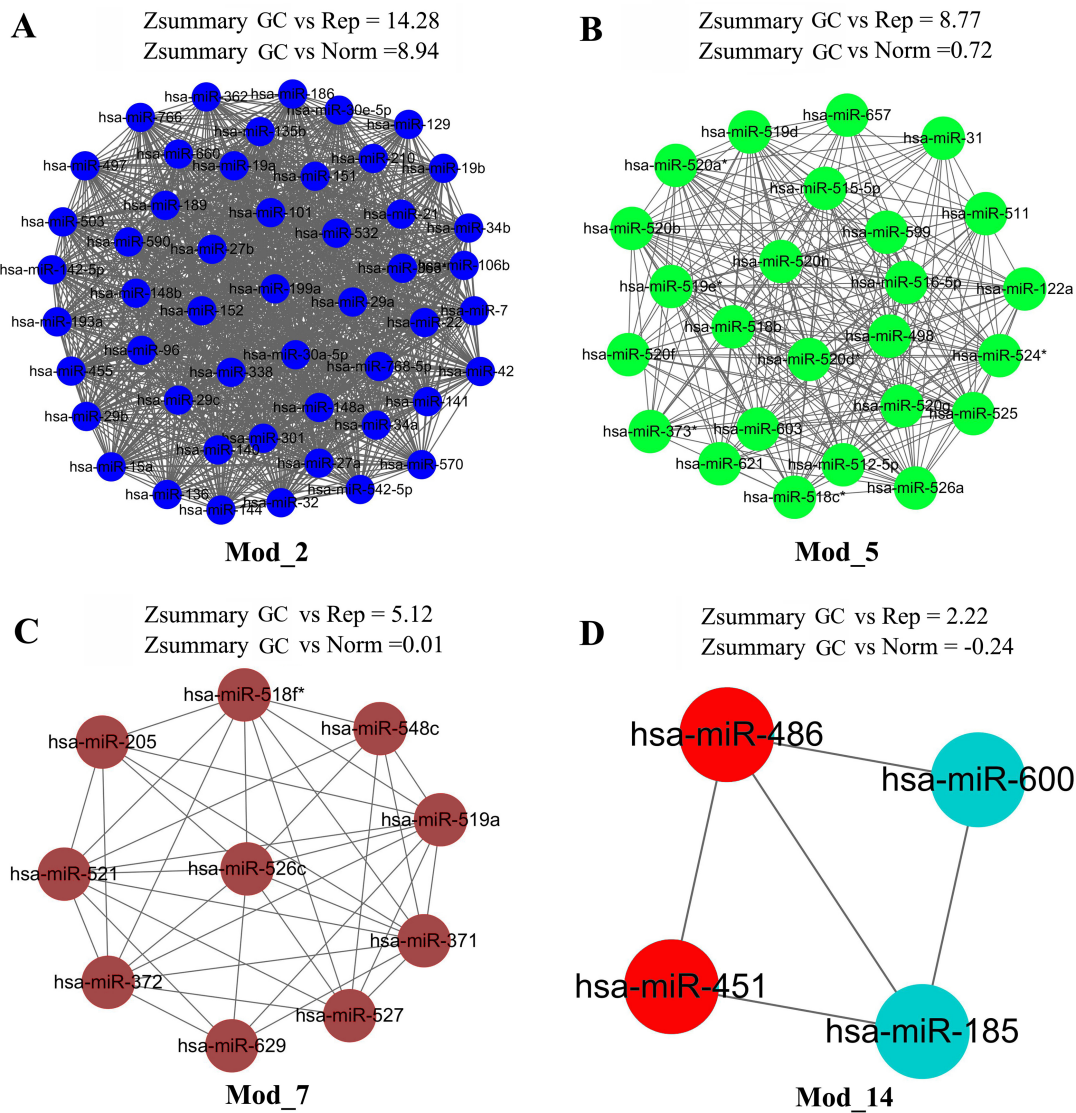
The representative modules with major variations of the Z_summary_ values. **A-C**. The preserved modules Mod_2, Mod_5, and Mod_7. **D**. The activated module (biomarker module) Mod_14; the two red nodes miR_486 and miR_451 are included in the list of the top 20 up-regulated miRNAs.

Compared with the Norm group, disruption or rewiring of the miRNA co-expression modules in the GC group might reflect the pathogenesis of GC. We still used Z_summary_ to assess the disruption of miRNA modules. Modules with a negative Z_summary_ were considered as the activated modules of GC. **Fig 2B** lists the Z_summary_ values of all the miRNA modules in the GC group compared with the Norm group. Two modules, i.e. Mod_14 and Mod_16, had negative Z_summary_ values, but Mod_16 was activated module in the GC v.s. Rep comparison, so only Mod_14 (**Fig 3D**) was chosen as the activated module of GC v.s. Norm, which was viewed as a potential miRNA module biomarker for GC. Modules with major variations of the Z_summary_ values when the GC group was compared with the Norm group or Rep group are also visualized in **Fig 3**.

### NetSVM-based core miRNAs and sub-network

**Fig 4:**
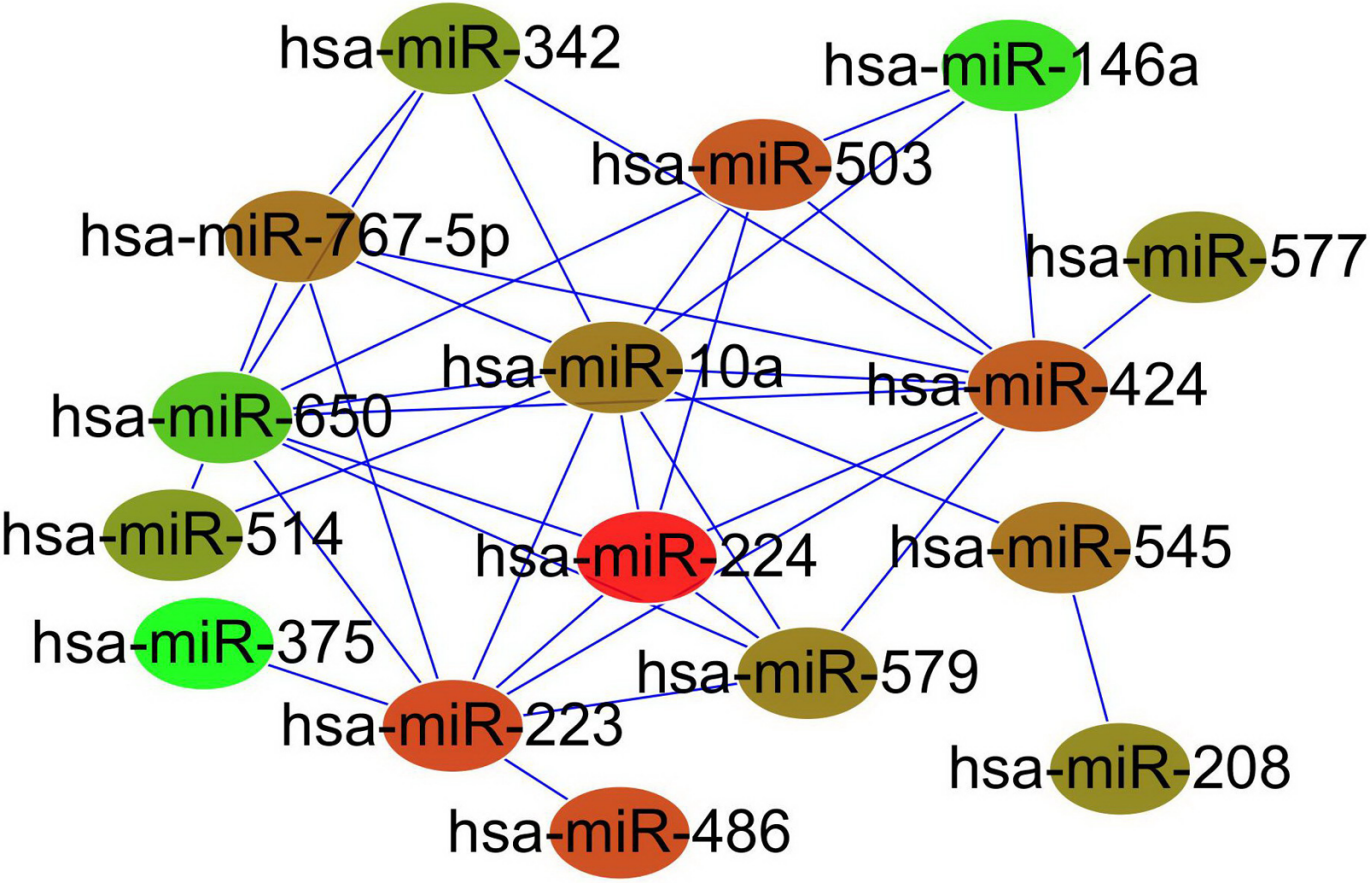
The sub-network comprised of the top 20 up- and down-regulated miRNAs. The node color (red denotes up-regulation and green denotes down-regulation) is set based on the log2 fold change of expression between GC and Norm groups.

Based on the miRNA expression levels and network interactions, the NetSVM [24] was used to identify the core miRNAs and sub-network as the potential GC biomarkers. Compared with the Norm group, the weights of all the miRNAs in networks were obtained (**S4 Table**). The top 20 up- or down-regulated miRNAs and their attributed modules are listed in **Table 1**. Among the top 40 miRNAs, MiR-424 and MiR-146a had the largest weights in the up- and down-regulated miRNAs, respectively; and 70% of these miRNAs belonged to a specific module. The members of the biomarker module (Mod_14), miR-486 and miR-451, ranked 14 and 20 of the up-regulated miRNAs, respectively (**Fig 3**). Taken the up- and down-regulated miRNAs together, a sub-network of the top 20 miRNAs (13 up-regulated and 7 down-regulated) was constructed (**Fig 4**), which had a high sensitivity and specificity of 0.94 and 0.82, respectively.

**Table 1:** The top 20 miRNAs and their attributed modules based on the NetSVM weights.

Up-regulated miRNAs	SVM weights	Attributed modules	Down-regulated miRNAs	SVM weights	Attributed modulse
**hsa-miR-424**	**0.2001**	Mod_2	**hsa-miR-146a**	**-0.2201**	Mod_1
**hsa-miR-503**	**0.1984**	Mod_2	**hsa-miR-376a**	**-0.2149**	Mod_3
**hsa-miR-224**	**0.1888**	Mod_1	**hsa-miR-647**	**-0.1615**	/
**hsa-miR-342**	**0.1794**	Mod_11	**hsa-miR-650**	**-0.1605**	Mod_1
**hsa-miR-767-5p**	**0.1586**	Mod_11	**hsa-miR-375**	**-0.1514**	Mod_8
**hsa-miR-577**	**0.1575**	/	**hsa-miR-504**	**-0.1491**	/
**hsa-miR-486**	**0.1570**	**Mod_14**	**hsa-miR-514**	**-0.1467**	/
**hsa-miR-10a**	**0.1545**	Mod_1	hsa-miR-625	-0.1430	Mod_1
**hsa-miR-545**	**0.1542**	/	hsa-miR-642	-0.1392	/
**hsa-miR-548d**	**0.1531**	Mod_16	hsa-miR-517	-0.1335	/
**hsa-miR-208**	**0.1529**	/	hsa-miR-660	-0.1277	Mod_2
**hsa-miR-223**	**0.1519**	Mod_1	hsa-miR-365	-0.1250	Mod_1
**hsa-miR-579**	**0.1485**	/	hsa-miR-203	-0.1231	Mod_8
hsa-miR-635	0.1445	Mod_1	hsa-miR-133a	-0.1231	Mod_13
hsa-miR-320	0.1439	/	hsa-miR-299-5p	-0.1225	Mod_3
hsa-let-7i	0.1433	Mod_1	hsa-miR-661	-0.1223	/
hsa-miR-582	0.1383	Mod_3	hsa-miR-95	-0.1213	Mod_1
hsa-miR-326	0.1359	/	hsa-miR-629	-0.1197	Mod_7
hsa-miR-484	0.1353	Mod_4	hsa-miR-155	-0.1146	Mod_1
hsa-miR-451	0.1296	**Mod_14**	hsa-miR-551b	-0.1143	/

The bold letters represent the top 20 miRNAs when both up- and down-regulated miRNAs are taken together.

### The diagnostic performance of the GC module biomarker

**Fig 5:**
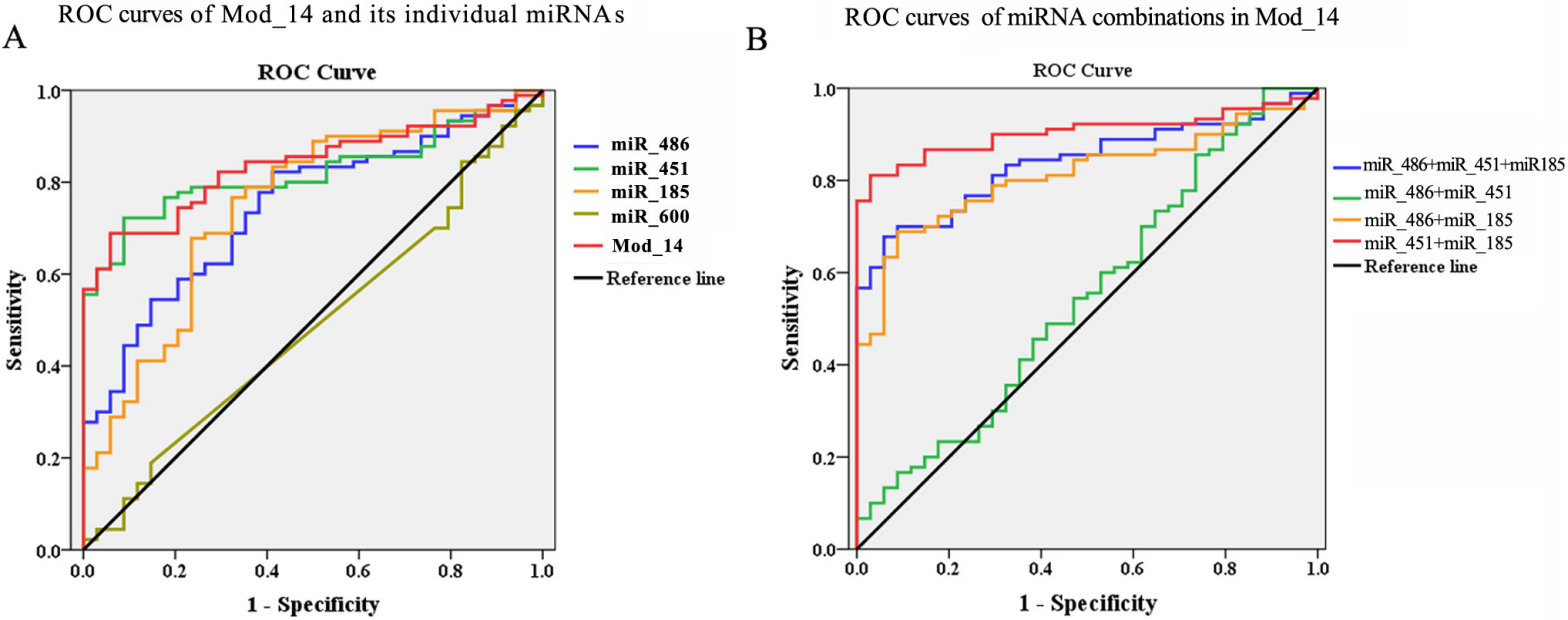
The diagnostic performance of the biomarker module Mod_14 and its individual miRNAs or combinations based on the ROC curves. **A**. ROC curves of Mod_14 and its individual miRNAs. Red line, Mod_14 (AUC = 0.838, sensitivity = 68.9%, specificity = 94.1%); Blue line, miR_486 (AUC = 0.747, sensitivity = 82.2%, specificity = 58.8%); Green line, miR_451 (AUC = 0.819, sensitivity = 72.2%, specificity = 91.2%); Orange line, miR_185 (AUC = 0.751, sensitivity = 76.7%, specificity = 67.6%); Greenyellow line, miR_600 (AUC = 0.487, sensitivity = 18.9%, specificity = 85.3%). **B.** ROC curves of miRNA combinations in Mod_14. Red line, combination of miR_451 and miR_185 (AUC = 0.904, sensitivity = 81.1%, specificity = 97.1%); Blue line, combination of miR_451, miR_486 and miR_185 (AUC = 0.838, sensitivity = 67.8%, specificity = 94.1%); Green line, combination of miR_451 and miR_486 (AUC = 0.550, sensitivity = 85.6%, specificity = 26.5%); Orange line, combination of miR_486 and miR_185 (AUC = 0.811, sensitivity = 68.9%, specificity = 91.2%).

The diagnostic performance of the biomarker Mod_14 and its miRNA members were evaluated based on the ROC AUC value (**Fig 5A, S5 Table**). The AUC value of the biomarker Mod_14 was 0.838, with optimal specificity of 0.941 and sensitivity of 0.689 (Youden's index = 0.63) at the cut-off value of 0.241, which was much higher than that of any individual miRNAs. Besides, we also evaluated the diagnostic performance of the combinations of certain Mod_14 members (**Fig 5B, S5 Table**). We found that the combination of miR-451 and miR-185 reached an even higher AUC value of 0.904 (Youden's index = 0.782) at the cut-off value of 0.26, with a sensitivity of 0.811 and a specificity of 0.971.

### Target genes and biological functions of the miRNA module biomarker

**Fig 6:**
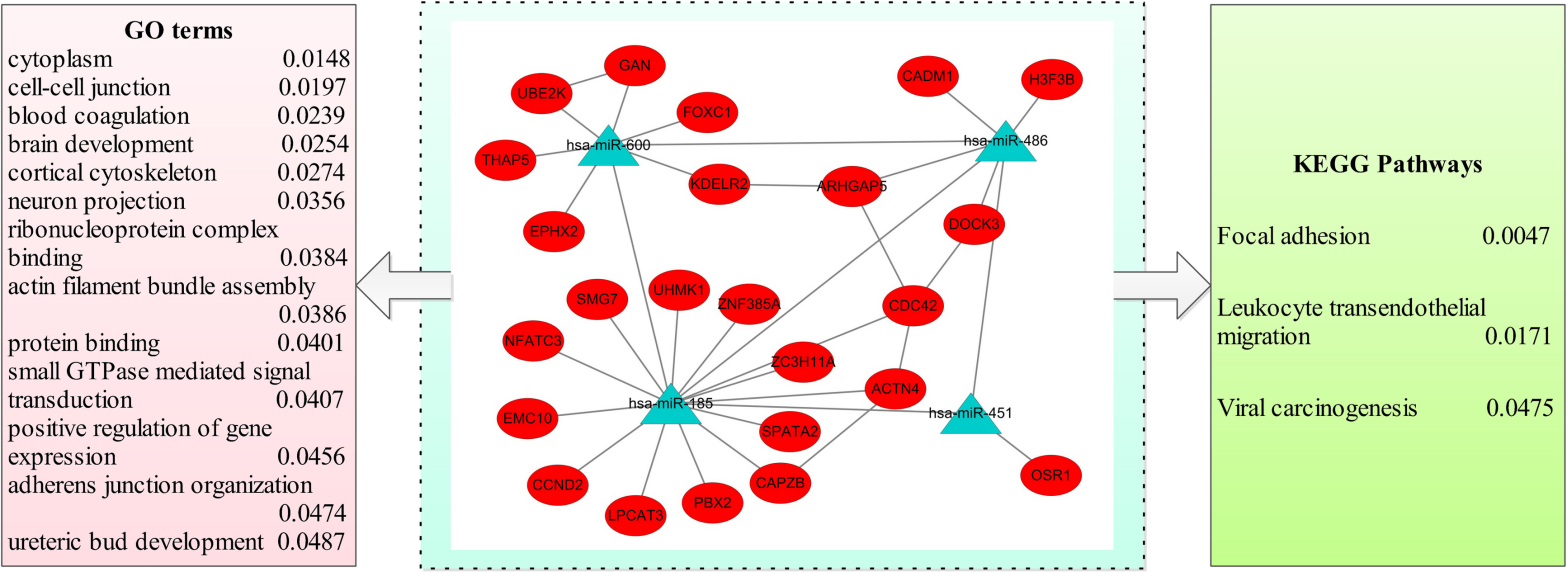
The target gene network and biological functions of the biomarker module Mod_14. The triangular labels represent miRNAs, and the elliptic labels represent target genes. The significantly enriched GO terms and pathways are listed with p values.

The target genes of the biomarker Mod_14 were predicted based on three databases. As mentioned in the Methods section, only those that were predicted by all of the three databases were considered as target genes. A total of 24 target genes of Mod_14 were obtained, of which 4, 1, 6 and 13 genes belonged to miR-486, miR-151, miR-600 and miR-185, respectively. The network of Mod_14 miRNAs and their target genes are shown in **Fig 6**.

To characterize the biological functions of the identified biomarker module Mod_14, we obtained its GO functions and pathways through the target gene enrichment analysis. The significantly enriched GO terms and pathways with their p values are listed in **Fig 6**. As shown in **Fig 6**, the most significantly enriched pathway and Go term were focal adhesion and cytoplasm, and pathways directly related to cancer were also found, such as the viral carcinogenesis pathway.

## Discussion

There is increasing evidence that the occurrence of complex diseases, including cancers, may be attributed to the perturbation of complex molecular networks, and both diseases and drug actions have a modular basis [26–27]. Module-based methods have promoted the discovery of cancer biomarkers and drug targets [28–29]. In this study, with the use of a Z_summary_-based method, a GC miRNA module biomarker was identified from the miRNA co-expression network, and the hub miRNAs and sub-network related to GC patients were also determined by the NetSVM algorithm. The target genes of the GC miRNA module biomarker and its functions were predicted based on multiple databases. By way of the modular analysis of GC miRNA and their targeted genes, we may light the further insight of GC diagnostics and therapy at multi-target level.

Within the identified miRNA module biomarker Mod_14 (consisting of miR-486, miR-451, miR-185, and miR-600), three of its miRNA members were over-expressed in GC patients [19], and thus this module was an up-regulated module for GC. Previous miRNA microarray studies revealed that miR-486 could regulate tumor progression and the OLFM4 antiapoptotic factor in GC [30]. Several studies indicated that miR-486 and miR-451 might act as novel prognostic biomarkers and potential therapeutic targets in human GC [31–32]. Moreover, a prior study reported that miR-451 and miR-486 showed consistently elevated levels in the plasma of GC patients as compared with controls [33]. As for miR-185, it has been shown that it may suppress tumor metastasis, regulate chemotherapeutic sensitivity, and serve as an independent prognostic factor for GC [34–35]. Therefore, three out of the four miRNA nodes in this module have been reported to have close relationship with GC, and these findings were largely consistent with our results. In terms of the biological functions, pathways and GO terms directly related to cancer were significantly enriched in this module, such as the viral carcinogenesis pathway.

In addition to the activated module biomarker, the hub miRNAs and sub-network for GC were also determined based on the NetSVM algorithm. The activated module nodes miR-486 and miR-451 were both included in the list of the top 20 up-regulated miRNAs. MiR-424 which had the largest weight in the up-regulated miRNAs was reported to promote the proliferation of gastric cancer by targeting Smad3 via TGF-u signaling pathway [36]. MiR-146a that had the largest weight in the down-regulated miRNAs may acts as a metastasis suppressor in GC by targeting WASF2 [37]. Besides, most of the top ranked miRNAs were members of a specific module, indicating that these miRNAs not only have great weights for GC, but also have close interactions with one another. Thus, this integrated network analysis may be effective in screening cancer-related miRNAs or sub-networks.

In conclusion, based on integrated network analysis, this study identified an activated miRNA module biomarker of GC, which was composed of miR-486, miR-451, miR-185, and miR-600. The hub miRNAs and sub-network of GC were also detected by a NetSVM-based method. These identified hub miRNAs and modules have the potential to become robust biomarkers for the diagnosis of early stage GC, although further validations are still required. Moreover, such a modular analysis of miRNA networks may offer valuable insights into multi-target approaches to cancer diagnosis and treatment.

## Supporting information

S1 Fig The scale-free topology model and mean connectivity of GC group datasets. (JPG)

S2 Fig The network modular hotmaps of all genes in GC group. (JPG)

S1 Table The miRNA expression data and detailed membership of modules in GC group. (CSV)

S2 Table The miRNA expression data of Rep group. (CSV)

S3 Table The miRNA expression data of Norm group. (CSV)

S4 Table The NetSVM-based weights of miRNAs in GC group. (CSV)

S5 Table The diagnostic value of combined and single miRNA markers for GC. (XLS)
