# Supplementary figures and images for: Optimizing miRNA-module diagnostic biomarkers of gastric carcinoma via integrated network analysis
Source: PLoS One. 2018 Jun 7;13(6):e0198445. doi: 10.1371/journal.pone.0198445 (PMC5991748; doi:10.1371/journal.pone.0198445)

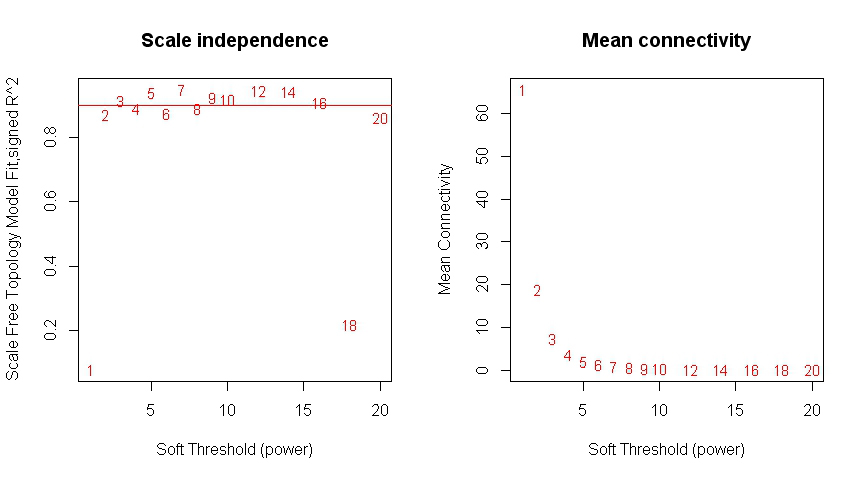

Supplement: S1 Fig — (JPG) [file pone.0198445.s001.jpg]

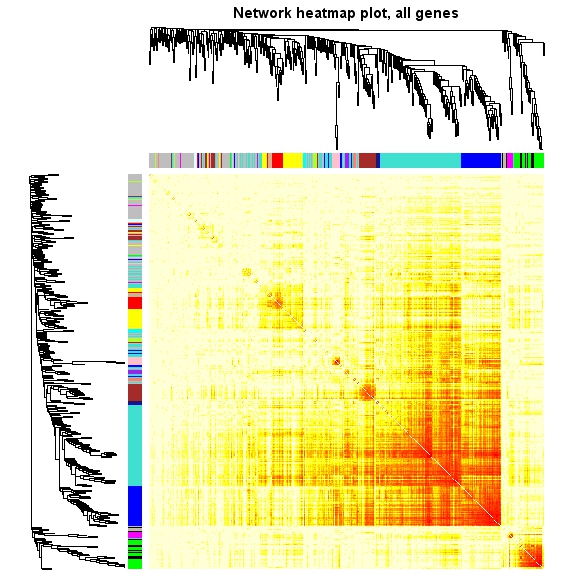

Supplement: S2 Fig — (JPG) [file pone.0198445.s002.jpg]
